# Sample‐Efficient Transfer Learning for Histopathological Detection of HNSCC

**DOI:** 10.1111/jop.70181

**Published:** 2026-08-31

**Authors:** Till Nicke, Fleming Wolf‐Eli Michelsen, Jan‐Raphael Schäfer, Jonas Wuester, Marc Christian Metzger, Rainer Schmelzeisen, Gian Kayser, Andreas Weber‐Steinhilber, Joachim Georgii, Johannes Lotz, Leonard Simon Brandenburg

**Affiliations:** ^1^ Fraunhofer Institute for Digital Medicine MEVIS Bremen Germany; ^2^ Medical Center—University of Freiburg, Department of Oral and Maxillofacial Surgery, Faculty of Medicine University of Freiburg Freiburg Germany; ^3^ Institute of Pathology Naehrig Mattern Kayser Freiburg Germany; ^4^ Institute for Surgical Pathology, Medical Center—University of Freiburg, Faculty of Medicine University of Freiburg Freiburg Germany; ^5^ Core Facility for Histopathology and Digital Pathology, Medical Center University of Freiburg Freiburg Germany

## Abstract

**Background:**

Head and neck squamous cell carcinoma (HNSCC) is a common malignancy with increasing incidence. Histopathological assessment remains the diagnostic gold standard, but is labor‐intensive and affected by a growing shortage of specialized pathologists. Limited availability of annotated data poses a major challenge for AI‐based support tools in digital pathology.

**Methods:**

We evaluated the transfer performance of a domain‐specific foundation model, Tissue Concepts, compared to standard ImageNet pretraining for HNSCC detection in histopathological whole slide images. A multicentric, retrospective dataset comprising digitized H&E‐stained slides from The Cancer Imaging Archive was used (390 slides, 268 HNSCC cases, 112 patients). Tumor regions were annotated by expert pathologists. Model representations were assessed without task‐specific fine‐tuning using a standardized classifier probe. Sample efficiency was investigated on patch‐based and patient‐based levels to estimate the number of annotated cases required for robust performance.

**Results:**

The foundation model consistently outperformed ImageNet pretraining across all experiments. On a patch‐based level, Tissue Concepts achieved substantially higher discrimination performance with minimal training data (AUROC = 0.70 vs. = 0.50 using only 1 patch). On a patient‐based level, the foundation model reached robust performance with a limited number of annotated cases (AUROC = 0.90 with 10 cases), whereas ImageNet‐based representations showed slightly inferior performance despite substantially larger training sets (AUROC = 0.87 with 50 cases). Performance improvements plateaued beyond 10 cases, indicating diminishing returns with additional annotations.

**Conclusion:**

Domain‐specific foundation models enable competitive HNSCC detection under annotation‐scarce conditions. However, performance depends on the representativeness of annotated cases and tumor heterogeneity across anatomical sites and institutions. Further multicenter and prospective studies are required to assess generalizability and integration into routine histopathological workflows.

## 1 Introduction

Head and neck malignancies are the sixth most commonly diagnosed type of cancer worldwide [1]. Head and neck squamous cell carcinoma (HNSCC) has an increasing incidence, especially in younger patients, making it a global health issue [2, 3].

An important cornerstone in the diagnosis of tumors is histopathological analysis, which is based on the light microscopic examination of stained tissue samples by a pathologist. Correct evaluation, especially in the early stages, is important for treatment selection and prognosis [4, 5, 6]. Histopathological diagnosis of HNSCC is challenging due to its variable cellular appearance, compared to other tumors, and therefore the domain of specialized pathologists [7]. In addition to inter‐patient variability, HNSCC exhibits pronounced morphological heterogeneity across anatomical sites, with tumor appearance strongly influenced by surrounding tissue structures [8]. This heterogeneity poses a challenge for data‐driven models, particularly when only limited annotated cases are available. Therefore, microscopic examination of specimens is time‐consuming, costly, and laborious, but also susceptible to human errors [5]. Furthermore, despite the rising incidence of cancer, there is an increasing shortage of pathologists [9].

As a result, there is a growing interest in automated solutions, such as digital or computer‐assisted pathology, to support pathologists in their work. Deep Learning (DL), which mimics the learning process of the human brain, has proven to be a promising support for increasing the efficiency and precision of histopathological diagnostics [10, 11]. Despite the remarkable progress in this field, research faces several challenges, such as the necessary modernization and digitalization of histopathological institutes. However, the most significant challenge in the development of DL models is the lack of annotated training data for algorithms to unfold their potential [12].

To effectively train a DL model, large amounts of data are required. Unfortunately, the limited amount of suitable medical data paired with strict data protection regulations results mostly in small datasets being available for DL training. Foundation Models (FM) offer a solution where robust transfer learning takes place using pretraining strategy based on either self‐supervision or supervised signals in the form of classification or segmentation [13]. A popular FM that combines multiple supervised signals using multitask learning (MTL) is the open‐source model Tissue Concepts [14, 15].

The present work addresses the challenge of limited annotated data in the development of deep learning–based support tools for histopathological HNSCC analysis. Rather than proposing a task‐specific diagnostic model, we investigate the transfer performance of a domain‐specific foundation model that has not been exposed to HNSCC tissue during pretraining. Using a standardized classifier probe, we compare representations derived from the foundation model with those obtained from ImageNet pretraining to assess how effectively tumor‐related features can be extracted under annotation‐scarce conditions. To this end, model performance is evaluated on both patch‐based and patient‐based levels, with a particular focus on estimating the number of annotated cases required to achieve robust and clinically meaningful performance.

## 2 Materials and Methods

In a multicentric, retrospective study, the training success in detecting tumor areas in whole slide images (WSI) of HNSCC was evaluated based on two different models. On the one hand, the FM Tissue Concepts [14, 15], that was previously pretrained on a combination of several medical datasets with different labeling types (see below). On the other hand, the same model architecture with ImageNet weights [16] was used as a baseline. The key difference is that the baseline underwent training with natural images, such as boat, house, animal, and so forth (see below), which still represents a reliable starting point in computational pathology [17].

### 2.1 Ethical Clearance

This study was conducted in accordance with the Helsinki Declaration and received ethical approval from the Ethics Committee of the Albert‐Ludwigs‐Universität of Freiburg (No. 23‐1105_1‐S1‐retro).

### 2.2 Data

Digitized histopathological sections from the publicly accessible archive “The Cancer Imaging Archive” (https://www.cancerimagingarchive.net), serve as the basis for data acquisition. This dataset comprises hematoxylin and eosin (H&E) stained slides of head and neck tissues, encompassing a total of 390 whole slide images (WSI). Of these, 268 were confirmed by a pathologist to contain HNSCC, while 122 were found to be tumor‐free. These sections originate from the treatment of 112 patients and were downloaded as Version 10. Table 1 gives an overview of the 268 slides with corresponding origins of regions.

**TABLE 1:** Distribution of tumor localizations in the HNSCC dataset from “The Cancer Imaging Archive”.

Number of WSIs (total: 268)	Depicted region
123	Larynx
51	Tongue
28	Floor of mouth
12	Lip
12	Oral cavity
10	Alveolar ridge
10	Oropharynx
9	Buccal mucosa
6	Base of tongue
3	Pyriform sinus
2	Epiglottis
2	Tonsil

For annotation, parts of the images where HNSCC was recognizable were marked within all 268 tumor‐containing WSI. This was done with the open‐source program QuPath, version 0.5.0 [18], using mainly the tools “wand” and “brush”. The annotations were continuously monitored by a pathologist with over 15 years of experience in the field. Figure 1 shows an example of the marked annotations.

**FIGURE 1:**
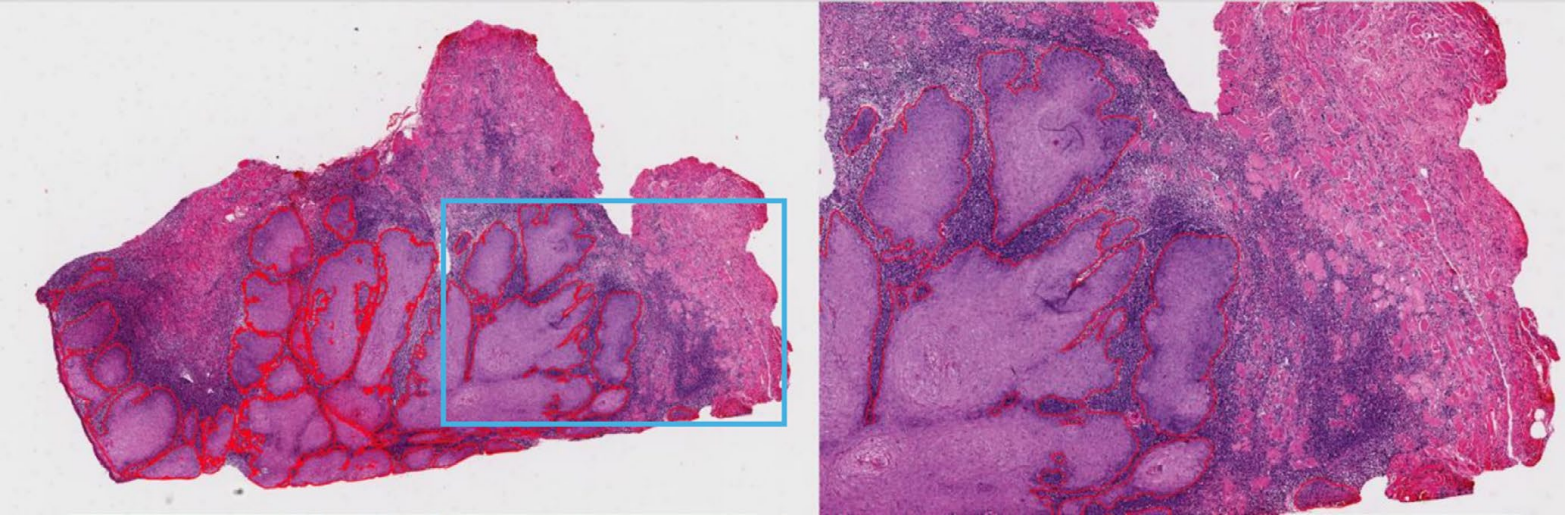
Example for annotations. Labeled tissue of a squamous cell carcinoma of the buccal mucosa. The red line frames regions with present carcinoma.

Small patches of 224 × 224 pixels were extracted from each WSI at approximately 0.5 μm per pixel (×20 magnification). Areas of the 268 whole slide images that were confirmed to show HNSCC were used as examples. Patches were considered positive examples of HNSCC when all pixels were placed within the annotated region. Patches were considered to show normal tissue if they were placed within the overall tissue area, but completely outside the annotated region. A total of 33,737 patches were extracted from all WSI. The data was divided into training, validation, and test sets at patient level.

### 2.3 Neural Networks

#### 2.3.1 Tissue Concepts

The foundation model Tissue Concepts [14, 15] is a universal model for biomedical images that has been pretrained with tomographic, microscopic, and radiologic images, which were each labeled with different strategies. The version of Tissue Concepts used is extended from the version published by [15] for better coverage of histopathology data. It was not previously trained on images showing HNSCC, but on a variety of different tissues and malignancies, giving the model inherent medical knowledge. See [14, 15] for further information regarding the training data and process. The architectural backbone of the model used in this study is based on a Swin‐Transformer v2 [19], with around 88M parameters. The model weights that were used in this study can be downloaded from the corresponding GitHub code repository (https://github.com/FraunhoferMEVIS/MedicalMultitaskModeling, version 1.0.5).

#### 2.3.2 Baseline—ImageNet

The baseline DL model, of the same architecture as Tissue Concepts, was previously pretrained on ImageNet. This pretraining dataset consists of thousands of nonmedical images, and the network learns to distinguish between different classes such as boat, house, animal, and so forth. Nonetheless, it is a relevant standard model in object recognition, detection, and classification in the medical imaging domain. In many studies regarding medical images, ImageNet weights serve as a starting point for further experiments [17]. The weights for the model were obtained from PyTorch (version 2.1.2).

### 2.4 Measuring Transfer Performance of Neural Networks

Transfer capabilities for both models were evaluated with respect to their sample efficiency, which measures how well a model performs with small amounts of data. The evaluation used unchanged model weights to assess performance under conditions of limited training data. Although ImageNet weights provide pretraining with natural images as a starting point, deep learning methods still require extensive annotated data to achieve robust results in medical imaging. To estimate how many annotated data points would be needed to train a robust classifier for tumor detection, random forests from the scikit‐learn library (version 1.5.2) were trained on the intermediate outputs of the models, effectively measuring their ability to extract meaningful and robust features from the input images. The models used in this study were specifically chosen due to their lack of exposure to HNSCC data, ensuring that the results would reflect performance in an unseen clinical setting. To extract features from the images, each patch was passed through the model's frozen backbone and then averaged along the height and width to obtain its latent representation, a single vector with 1024 numerical entries. No further preprocessing of the patches was used to best mimic clinical routine. Each vector was written to disk and loaded to be used as input to the random forest classifier. No further fine‐tuning or training of the artificial neural network occurred during these experiments, allowing for a direct assessment of each model's capability to extract robust and general features from the images.

#### 2.4.1 Patch‐Based Transfer Tasks

Sample efficiency tests to measure patch‐based transfer involved training a random forest classifier with varying numbers of annotated patches per class (1, 4, 8, 16, 32, 64, 128, 512, 1024, 2048, and 4096 patches). Each configuration was repeated 10 times to account for randomness. The objective was to distinguish between patches from HNSCC annotated regions and those from nonannotated regions. The performance was evaluated using balanced accuracy, F1‐score, and AUC. For patch‐based transfer, a fixed, balanced test set of 800 patches that were excluded from training was used.

#### 2.4.2 Patient‐Based Transfer Tasks

For patient‐based transfer capabilities, patients were randomly selected to be within the training set. Patients that were not randomly chosen as training were used in the test set. Fixed random seeds were used to ensure fair comparisons between the models. To train the random forest classifier, all patches originating from the same patient were used as either training or testing patches. During training, all patches from a specific patient were sampled and concatenated with all other patches from patients selected in the training set. These concatenated vectors were then used as input to the random forest classifier to learn a differentiation between patches from annotated HNSCC regions and not annotated regions (cancerous vs. noncancerous). During testing, the individual patient performance was measured as balanced accuracy and AUROC of the random forest classifier on all patches of that patient. To obtain a score for an individual patient, the performance over all patches from that patient was averaged. The experiment was repeated five times to account for randomization artifacts, and each time, 1, 3, 5, 10, 20, 30, 40, and 50 patients were selected as training patients.

## 3 Results

### 3.1 Patch‐Based Transfer Tasks

Both models (Tissue Concepts and ImageNet) showed an increase in performance with more images per class used to train the random forest. A significant difference in performance between the networks was observed. Tissue Concepts achieved higher accuracy and AUC compared to ImageNet based on less training data. Figure 2 provides an overview of the test AUC as a function of the underlying training data for patch‐based training.

**FIGURE 2:**
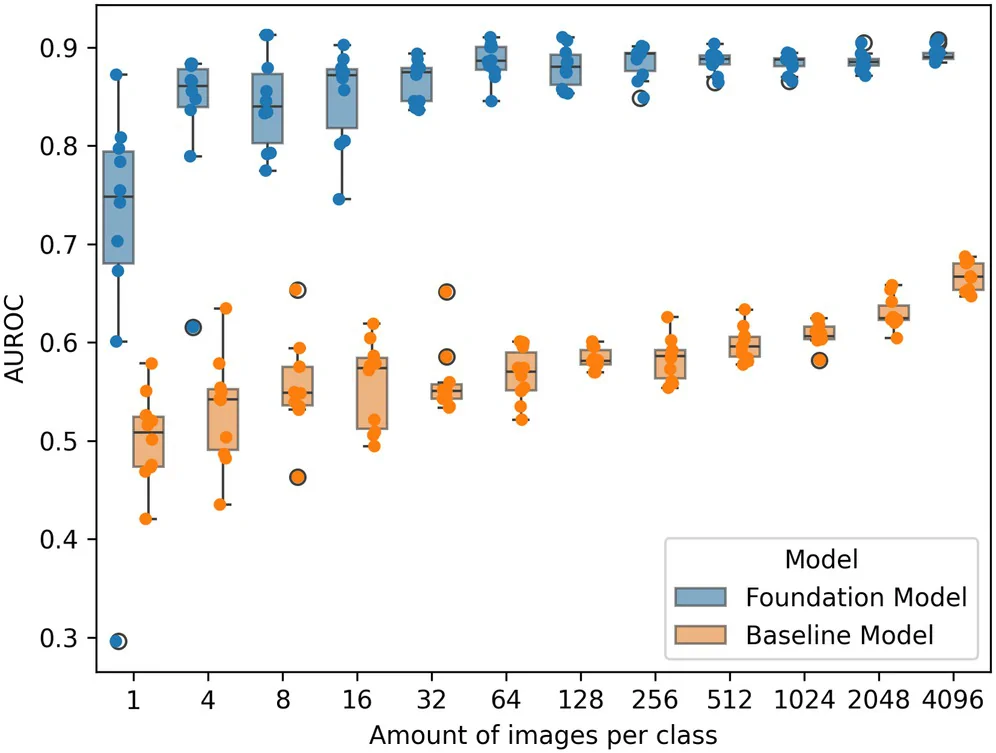
Results for different numbers of patches per class using Tissue Concepts (FM) and ImageNet (baseline). The different numbers of patches per class are plotted on the x‐axis against the test AUROC score on the y‐axis. The box plots show the interquartile range, with each point representing a score from a particular run.

The FM Tissue Concepts outperforms the ImageNet baseline weights with only one patch per class, with an average AUROC of 0.71, while the baseline model achieves an AUROC of only 0.50. With more patches per class, both models perform better on the test set, but the FM achieves a higher AUROC with 1 patch per class (0.71) than the baseline model with 4096 images per class (0.67). The FM achieved its best performance with 4096 images per class, achieving an AUROC of 0.89 on the test set. However, performance did not improve drastically after 64 images per class. Additional metrics of all settings can be found in Table 2.

**TABLE 2:** Results for different number of patches per class displayed for the foundation model Tissue Concepts and ImageNet weights.

Number of patches (224 × 224 pixels)	Accuracy of tissue concepts (%)	F1‐score of tissue concepts	AUROC of tissue concepts	Accuracy of ImageNet (%)	F1‐score of ImageNet	AUROC of ImageNet
1	63.9 (±9)	74.1 (±16)	70.2 (±15)	53.9 (± 5)	56.9 (±7)	50.2 (±4)
4	72.5 (±10)	83.3 (±8)	83.2 (±8)	56.8 (±7)	40.0 (±10)	53.1 (±5)
8	80.1 (±8)	85.3 (±4)	84.3 (±4)	60.0 (±8)	46.2 (±9)	55.6 (±4)
16	83.3 (±4)	85.6 (±4)	84.9 (±3)	56.2 (±3)	44.3 (±6)	55.6 (±4)
32	85.5 (±2)	87.2 (±2)	86.5 (±2)	58.5 (±4)	45.7 (±5)	56.7 (±2)
64	86.2 (±1)	89.8 (±1)	88.5 (±1)	57.8 (±3)	46.5 (±5)	56.9 (±2)
128	87.5 (±1)	88.9 (±2)	87.9 (±1)	61.4 (± 2)	46.7 (±4)	58.3 (±1)
256	88.5 (±1)	89.0 (±1)	88.5 (±1)	63.9 (±1)	48.3 (± 3)	59.6 (±1)
512	88.8 (±1)	89.1 (±1)	88.5 (±1)	64.5 (±3)	49.4 (±3)	59.8 (±1)
1024	89.0 (±1)	88.9 (±1)	88.5 (±1)	66.5 (±3)	49.9 (±2)	60.7 (±1)
2048	89.5 (±0.7)	89.1 (±1)	88.7 (±1)	70.9 (±2)	53.5 (±3)	62.9 (±1)
4096	90.9 (±0.5)	89.2 (±1)	89.3 (±1)	75.4 (±1)	59.6 (±2)	66.9 (±1)

*Note:* Scores are test accuracy on a held‐out test set of 800 patches over 10 different runs, ± denotes the standard deviation over 10 runs.

### 3.2 Patient‐Based Transfer Tasks

The results of patient‐based transfer experiments show that, with 10 annotated patients, the FM reaches an average AUROC > 0.90. On average, ImageNet did not show an AUROC larger than 0.90 over all runs, with up to 50 patients in training. Figure 3 shows the increase in AUROC over the number of patients used during training. The red line marks 0.90, which is not crossed by the baseline model. An increase in performance can be seen with an increase in patients used during training for both models. Once 10 patients had been sampled, improvements in performance leveled off for both models, with no major enhancements observed for higher numbers of patients within the training.

**FIGURE 3:**
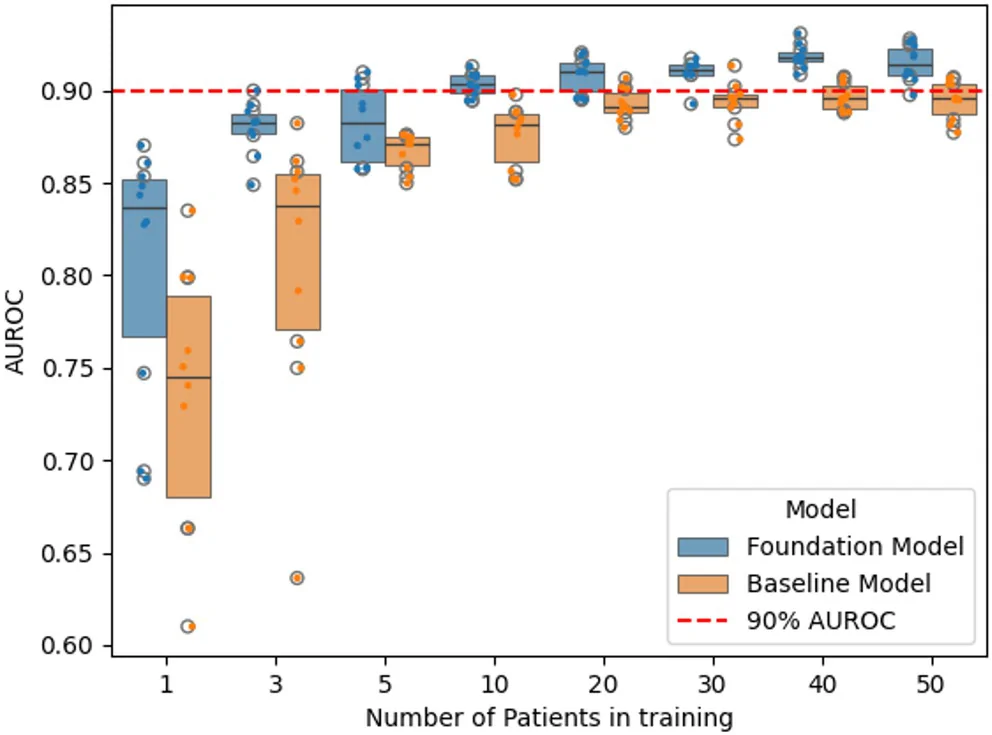
AUROC by patients used during training. Different numbers of patients were used to train a random forest classifier, while the rest were used as a holdout test set. 90% AUROC shown as a red cutoff line.

## 4 Discussion

This study demonstrates that a medical foundation model can extract transferable representations for histopathological HNSCC analysis under annotation‐scarce conditions. Without any task‐specific fine‐tuning and despite the absence of HNSCC tissue during pretraining, Tissue Concepts consistently outperformed ImageNet pretraining in both patch‐based and patient‐based transfer experiments. On a patch‐based level, the foundation model achieved higher discrimination performance with minimal training data, indicating improved sample efficiency of the learned representations. On a patient‐based level, competitive performance was reached with a limited number of annotated cases, although the performance gap between the foundation model and the baseline was smaller than observed in patch‐based experiments. Together, these findings suggest that medically pretrained foundation models may reduce the amount of annotation required to obtain competitive performance on previously unseen histopathological tasks.

The superior performance of Tissue Concepts compared to ImageNet pretraining can be attributed to more suitable feature representations for histopathological images, particularly when only limited annotated data are available, rather than to task‐specific optimization. Tissue Concepts was designed as a medically pretrained foundation model for computational pathology, which plausibly provides features that are more aligned with histomorphological patterns than representations learned from natural images, even when the target entity (HNSCC) was not part of pretraining. In line with the general motivation for foundation models under data scarcity, the patch‐based scaling curves suggest that medically pretrained representations are separable with very few training examples, whereas ImageNet features approach useful performance only with substantially larger sample counts. The plateauing of patch‐level performance beyond moderate sample sizes indicates diminishing returns from additional patches once the classifier probe has seen a sufficiently diverse set of appearances, which is consistent with prior discussions of limited‐data challenges in computational pathology and histopathology deep learning. Importantly, the smaller gap at the patient‐based level suggests that patient‐level aggregation partially mitigates patch‐level variability; once a limited number of annotated patients is available, both representations benefit from increased coverage of intra‐patient and inter‐patient variation, reducing the relative advantage of the foundation model. This interpretation is compatible with the broader computational pathology literature, where high HNSCC detection performance is commonly achieved after task‐specific training or fine‐tuning on curated datasets, typically starting from natural‐image pretrained models [17]. Recent studies report high accuracy for HNSCC detection on HE‐stained specimens, but they generally rely on extensive task‐specific optimization and do not explicitly quantify annotation‐efficiency at the patient level, which is the primary practical question addressed here [6, 7, 20, 21] (see Appendix A).

The results of this study have practical implications for the development of AI‐assisted tools in histopathological workflows, particularly with respect to annotation effort and diagnostic turnaround times. Manual annotation of histopathological whole slide images is time‐consuming and requires substantial expert involvement, representing a major bottleneck for the adoption of deep learning approaches in routine pathology [12, 17]. Delays in histopathological diagnosis are clinically relevant in HNSCC, as timely confirmation is critical for treatment planning and prognosis, especially given that many patients present at advanced disease stages [22, 23].

The observed patient‐based transfer performance suggests that competitive results can be achieved with a limited number of annotated cases, indicating that exhaustive large‐scale annotation may not be strictly necessary for initial model adaptation. This is particularly relevant in clinical environments facing increasing case numbers and a shortage of specialized pathologists, where reducing annotation effort may help support diagnostic workflows rather than replace expert judgment [10, 24]. From this perspective, foundation models may facilitate the rapid development of supportive tools for tasks such as prescreening, quality assurance, or prioritization of suspicious regions, complementing routine histopathological assessment.

Importantly, the diminishing performance gains observed beyond a limited number of annotated patients highlight that careful case selection is essential. Representative coverage of morphological variability may be more critical than increasing annotation volume alone, particularly in heterogeneous diseases such as HNSCC. This aligns with histopathological observations that oral squamous cell carcinoma exhibits pronounced intra‐ and intertumoral heterogeneity, which may limit the representativeness of small or morphologically narrow training cohorts [8] (see Appendix B). Such considerations support recent recommendations that emphasize annotation‐efficient, workflow‐aware AI systems over maximally optimized models as a more realistic path toward clinical translation [12, 25, 26].

Several limitations of this study should be acknowledged. First, the analysis is based on a retrospective dataset derived from The Cancer Imaging Archive. Although the dataset includes cases from multiple institutions, it may still be affected by dataset‐specific biases related to case selection, staining protocols, slide preparation, and digitization, which are well‐known challenges in computational pathology and can limit generalizability [12, 17]. Second, while patient‐based transfer analysis provides a more clinically meaningful perspective than patch‐based evaluation, the absolute number of patients remains limited, and performance estimates may depend on the representativeness of the selected cases. This is particularly relevant for HNSCC, which is characterized by pronounced morphological heterogeneity across patients and disease presentations [8].

Furthermore, the present study focuses on representation transfer using a fixed classifier probe and does not investigate end‐to‐end training or systematic fine‐tuning strategies. While this design choice enables a controlled assessment of sample efficiency and transfer performance, it does not capture the full performance potential achievable through task‐specific optimization, which is commonly employed in state‐of‐the‐art HNSCC detection approaches [6, 20, 27]. Finally, no prospective validation or evaluation within routine diagnostic workflows was performed. As emphasized in recent reviews, clinical translation of AI‐based pathology tools requires prospective, multi‐center studies and careful assessment of how algorithmic support interacts with pathologist decision‐making in real‐world settings [10, 17].

Future work should focus on validating the presented findings in prospective and multicenter settings to better assess robustness and generalizability under real‐world conditions. In particular, evaluating foundation model–based approaches across different institutions, staining protocols, and scanners will be essential to understand how annotation‐efficient transfer performance translates beyond retrospective benchmark datasets. Such studies should also consider a broader range of HNSCC subtypes and disease stages to more fully capture clinically relevant heterogeneity.

In addition, future investigations could explore how representation‐based approaches, as presented here, interact with task‐specific optimization strategies, including selective fine‐tuning or hybrid training schemes, to balance annotation effort, computational cost, and performance. Importantly, the integration of AI‐supported tools into routine histopathological workflows should be studied in close collaboration with pathologists, with a focus on usability, interpretability, and impact on diagnostic processes rather than isolated performance metrics. Addressing these aspects will be critical for translating annotation‐efficient foundation model approaches into clinically meaningful decision support systems.

## 5 Conclusion

In this study, we evaluated the transfer performance of a medical foundation model for histopathological HNSCC analysis under limited annotation. Without task‐specific fine‐tuning and despite the absence of HNSCC tissue during pretraining, the foundation model consistently outperformed ImageNet pretraining on both patch‐based and patient‐based transfer tasks. In particular, patient‐based transfer experiments indicated that competitive performance can be achieved with a limited number of annotated cases, while additional annotations resulted in diminishing performance gains.

These results suggest that medically pretrained foundation models may reduce the amount of annotation required to adapt AI‐based tools to previously unseen histopathological tasks. Rather than aiming to replace expert assessment, such approaches may support histopathological workflows by enabling annotation‐efficient development of task‐specific support tools.

Nevertheless, the findings of this study are subject to limitations related to dataset composition, case representativeness, and the retrospective nature of the analysis. Further prospective and multicenter studies are required to assess generalizability, robustness, and the role of foundation model–based approaches in routine histopathological practice.

## Author Contributions

**Fleming Wolf‐Eli Michelsen:** writing – original draft; writing – review and editing; data curation; **Jan‐Raphael Schäfer:** methodology; writing – review and editing; **Jonas Wuester:** writing – review and editing; **Marc Christian Metzger:** writing – review and editing; **Rainer Schmelzeisen:** writing – review and editing; **Gian Kayser:** writing – review and editing; data curation; supervision; **Andreas Weber‐Steinhilber:** writing – review and editing; **Joachim Georgii:** writing – review and editing; supervision; **Johannes Lotz:** writing – review and editing; supervision; **Leonard Simon Brandenburg:** writing – original draft; writing – review and editing; supervision; validation.

## Funding

This work was supported by Federal Ministry of Research, Technology and Space (BMFTR): 16SV9547.

## Ethics Statement

This study was conducted in accordance with the Helsinki Declaration and received ethical approval from the Ethics Committee of the Albert‐Ludwigs‐Universität of Freiburg (No. 23‐1105_1‐S1‐retro) – Head: Prof. Dr. R. Korinthenberg.

## Conflicts of Interest

The authors declare no conflicts of interest.

## Figures and Tables

**FIGURE B1:**
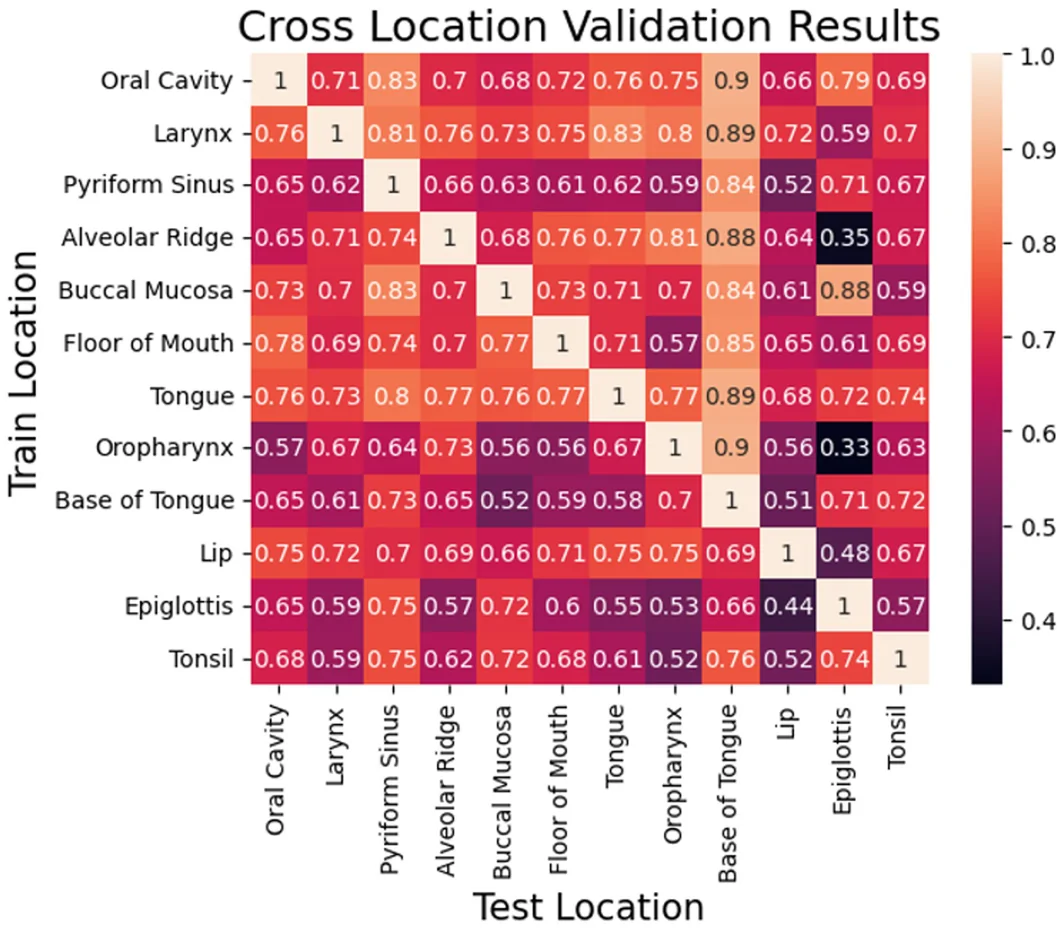
Matrix representing the different balanced accuracy scores of transferring learned representations from one location to another. Y‐Axis lists the “Train Location” vs. the “Test Location” on the X‐axis.

## Data Availability

The data used is freely available under https://portal.gdc.cancer.gov/analysis_page?app=CohortBuilder&tab=general. The weights of the models are also available under https://github.com/FraunhoferMEVIS/MedicalMultitaskModeling (version 1.0.5).

## Appendix A Fine‐Tuning on External Dataset

To obtain results comparable to the literature, the fine‐tuning capabilities of the FM were measured on an external validation dataset published by [28]. This dataset comprises 230 patients in a total of 1224 images at ×100 and ×400 magnification from 89 images of physiologic tissue and 439 images showing HNSCC. All images were captured using a camera mounted to a Leica ICC50 HD microscope from HE‐stained tissue slides. The FM was fine‐tuned for 15 epochs, with a learning rate of 1e‐5 using an AdamW optimizer [29] on the predefined training dataset. The best‐performing model was selected using the minimal validation loss over all epochs. This procedure was repeated 5 times to account for randomization artifacts.

An average AUC of 0.9994, average F1 of 0.9640, and average accuracy of 0.9804 were obtained on the test set of [28] with a magnification of ×100 over 5 runs. All results showed standard deviations of 0.01 or smaller. An overview of the performances with other approaches trained on the dataset of [28] can be seen in Table A1. All the methods compared exhibit strong results with accuracies above 95%. While there are some minor differences, these absolute values suggest that the FM as a base model is as performant as other methods, which require more extensive fine‐tuning. However, fine‐tuning the foundation model took less than 60 min of overall computation time. Additionally, a standard GPU (Nvidia A100) was sufficient to achieve these results.

**TABLE A1:** Comparison of the present study with recent publications on AI‐based detection of HNSCC in H&E‐stained specimens, published in 2024, on the benchmark dataset published in [28].

Publication	Accuracy	F1‐score	AUROC
Present study	0.9804	0.9640	0.9994
[21]	0.9875	0.9769	—
[6]	0.911	0.8654	—
[20]	0.90	0.93	0.96
[7]	0.9875	—	—
[30]	0.950	0.970	—
[27]	0.980	0.980	—

*Note:* A recent systematic review summarizes the publications on AI‐based HNSCC detection published before 2024 [17].

## Appendix B Cross‐Location Based Transfer

To estimate the transfer performance between different locations of HNSCC, the same dataset was used with a location‐based train and test distribution. For the cross‐location experiments, all WSIs of one location, tongue, lip, and so forth, were chosen once as the training set, and all other locations were chosen as the test set. A random forest classifier was trained to differentiate between patches originating from annotated regions and patches originating from nonannotated regions. By iterating through all locations and plotting the test accuracies (balanced) in a heatmap in Figure B1, transfer capabilities from one location to other locations can be visualized. As the foundation model significantly outperformed the ImageNet weights in the other transfer evaluations, ImageNet weights were not considered.

The results show that the location to which it is easiest to transfer is the base of tongue location, with an average balanced accuracy of 0.827. The most difficult location is the lip with an average balanced accuracy of 0.591. Looking at the heatmap in Figure B1, the test location lip (*x*‐axis) yields the worst results for almost every training location. The inverse is true for the test location base of tongue. Additionally, the epiglottis shows slightly worse transfer performance compared to other locations. This could be due to the three locations, which greatly underperform on the epiglottis. Alveolar ridge, oropharynx, and lip all result in bad test performances when applied to the epiglottis. These differences could potentially be due to the cartilage of the epiglottis, which differs in appearance from the soft tissue of other locations.

## References

[1] N. Cohen , S. Fedewa , and A. Y. Chen , “Epidemiology and Demographics of the Head and Neck Cancer Population,” Oral and Maxillofacial Surgery Clinics of North America 30, no. 4 (2018): 381–395, 10.1016/j.coms.2018.06.001. 30078696

[2] J. P. Machiels , C. R. Leemans , W. Golusinski , C. Grau , L. Licitra , and V. Gregoire , “Squamous Cell Carcinoma of the Oral Cavity, Larynx, Oropharynx and Hypopharynx: EHNS–ESMO–ESTRO Clinical Practice Guidelines for Diagnosis, Treatment and Follow‐Up,” Annals of Oncology 31, no. 11 (2020): 1462–1475, 10.1016/j.annonc.2020.07.011. 33239190

[3] G. Sarode , N. Maniyar , S. C. Sarode , M. Jafer , S. Patil , and K. H. Awan , “Epidemiologic Aspects of Oral Cancer,” Disease‐a‐Month 66, no. 12 (2020): 100988, 10.1016/j.disamonth.2020.100988. 32605720

[4] M. Fahad , M. U. Qayyum , and N. Abbasi , “AI in Histopathology: Automated Cancer Diagnosis to Detect Cancerous Cells and Assess Tumor Grade,” European Journal of Science, Innovation and Technology 3, no. 5 (2023): 5.

[5] S. Prabhu , K. Prasad , A. Robels‐Kelly , and X. Lu , “AI‐Based Carcinoma Detection and Classification Using Histopathological Images: A Systematic Review,” Computers in Biology and Medicine 142 (2022): 105209, 10.1016/j.compbiomed.2022.105209. 35042151

[6] A. Soni , P. K. Sethy , A. K. Dewangan , A. Nanthaamornphong , S. K. Behera , and B. Devi , “Enhancing Oral Squamous Cell Carcinoma Detection: A Novel Approach Using Improved EfficientNet Architecture,” BMC Oral Health 24, no. 1 (2024): 601, 10.1186/s12903-024-04307-5. PMC11112956 38783295

[7] E. Albalawi , A. Thakur , M. T. Ramakrishna , et al., “Oral Squamous Cell Carcinoma Detection Using EfficientNet on Histopathological Images,” Frontiers in Medicine 10, no. January (2024): 1349336, 10.3389/fmed.2023.1349336. PMC10859441 38348235

[8] G. Sarode , S. C. Sarode , J. Tupkari , and S. Patil , “Is Oral Squamous Cell Carcinoma Unique in Terms of Intra‐ and Inter‐Tumoral Heterogeneity?,” Translational Research in Oral Oncology 2, no. January (2017): 2057178X17703578, 10.1177/2057178X17703578.

[9] E. Walsh and N. M. Orsi , “The Current Troubled State of the Global Pathology Workforce: A Concise Review,” Diagnostic Pathology 19, no. 1 (2024): 163, 10.1186/s13000-024-01590-2. PMC11662708 39709433

[10] C. McGenity , E. L. Clarke , C. Jennings , et al., “Artificial Intelligence in Digital Pathology: A Systematic Review and Meta‐Analysis of Diagnostic Test Accuracy,” npj Digital Medicine 7, no. 1 (2024): 1–19, 10.1038/s41746-024-01106-8. PMC11069583 38704465

[11] I. Prassas , B. Clarke , T. Youssef , et al., “Computational Pathology: An Evolving Concept,” Clinical Chemistry and Laboratory Medicine (CCLM) 62, no. 11 (2024): 2148–2155, 10.1515/cclm-2023-1124. 38646706

[12] S. Morales , K. Engan , and V. Naranjo , “Artificial Intelligence in Computational Pathology – Challenges and Future Directions,” Digital Signal Processing 119, no. December (2021): 103196, 10.1016/j.dsp.2021.103196.

[13] M. J. Willemink , H. R. Roth , and V. Sandfort , “Toward Foundational Deep Learning Models for Medical Imaging in the New Era of Transformer Networks,” Radiology: Artificial Intelligence 4, no. 6 (2022): e210284, 10.1148/ryai.210284. PMC9745439 36523642

[14] T. Nicke , J. R. Schaefer , H. Hoefener , et al., “Tissue Concepts: Supervised Foundation Models in Computational Pathology,” Computers in Biology and Medicine 186, no. March (2025): 109621, 10.1016/j.compbiomed.2024.109621. 39793348

[15] R. Schäfer , T. Nicke , H. Höfener , et al., “Overcoming Data Scarcity in Biomedical Imaging With a Foundational Multi‐Task Model,” Nature Computational Science 4, no. 7 (2024): 495–509, 10.1038/s43588-024-00662-z. PMC11288886 39030386

[16] O. Russakovsky , J. Deng , H. Su , et al., “ImageNet Large Scale Visual Recognition Challenge,” International Journal of Computer Vision 115, no. 3 (2015): 211–252, 10.1007/s11263-015-0816-y.

[17] Z. Pirayesh , H. Mohammad‐Rahimi , N. Ghasemi , et al., “Deep Learning‐Based Image Classification and Segmentation on Digital Histopathology for Oral Squamous Cell Carcinoma: A Systematic Review and Meta‐Analysis,” Journal of Oral Pathology & Medicine: Official Publication of the International Association of Oral Pathologists and the American Academy of Oral Pathology 53, no. 9 (2024): 551–566, 10.1111/jop.13578. 39256895

[18] P. Bankhead , M. B. Loughrey , J. A. Fernández , et al., “QuPath: Open Source Software for Digital Pathology Image Analysis,” Scientific Reports 7, no. 1 (2017): 16878, 10.1038/s41598-017-17204-5. PMC5715110 29203879

[19] Z. Liu , H. Hu , Y. Lin , et al., “Swin Transformer V2: Scaling up Capacity and Resolution,” arXiv 2111 (2022): 09883, 10.48550/arXiv.2111.09883.

[20] K. Dhanya , D. V. V. Prasad , and Y. V. Lokeswari , “Detection of Oral Squamous Cell Carcinoma Using Pre‐Trained Deep Learning Models,” Experimental Oncology 46, no. 2 (2024): 2, 10.15407/exp-oncology.2024.02.119. 39396172

[21] M. Ragab and T. O. Asar , “Deep Transfer Learning With Improved Crayfish Optimization Algorithm for Oral Squamous Cell Carcinoma Cancer Recognition Using Histopathological Images,” Scientific Reports 14, no. 1 (2024): 25348, 10.1038/s41598-024-75330-3. PMC11512072 39455617

[22] L. Q. M. Chow , “Head and Neck Cancer,” New England Journal of Medicine 382, no. 1 (2020): 60–72, 10.1056/NEJMra1715715. 31893516

[23] J. J. Sciubba , “Oral Cancer,” American Journal of Clinical Dermatology 2, no. 4 (2001): 239–251, 10.2165/00128071-200102040-00005. 11705251

[24] S. M. H. Ali , U. M. Kathia , M. U. M. Gondal , A. Zil‐E‐Ali , H. Khan , and S. Riaz , “Impact of Clinical Information on the Turnaround Time in Surgical Histopathology: A Retrospective Study,” Cureus 10, no. 5 (2018): e2596, 10.7759/cureus.2596. PMC6037332 30009108

[25] A. Obeid , S. Boumaraf , A. Sohail , et al., “Advancing Histopathology With Deep Learning Under Data Scarcity: A Decade in Review,” arXiv (2024): 2410.19820, 10.48550/arXiv.2410.19820.

[26] X.‐M. Zhang , T.‐H. Gao , Q.‐Y. Cai , et al., “Artificial Intelligence in Digital Pathology Diagnosis and Analysis: Technologies, Challenges, and Future Prospects,” Military Medical Research 12, no. 1 (2026): 93, 10.1186/s40779-025-00680-6. PMC12765299 41484927

[27] M. Das , R. Dash , S. K. Mishra , and A. K. Dalai , “An Ensemble Deep Learning Model for Oral Squamous Cell Carcinoma Detection Using Histopathological Image Analysis,” IEEE Access 12 (2024): 127185–127197, 10.1109/ACCESS.2024.3450444.

[28] T. Y. Rahman , L. B. Mahanta , A. K. Das , and J. D. Sarma , “Histopathological Imaging Database for Oral Cancer Analysis,” 2023, 2 (January), 10.17632/ftmp4cvtmb.2. PMC6994517 32021884

[29] I. Loshchilov and F. Hutter , “Decoupled Weight Decay Regularization,” arXiv 1711 (2019): 05101, 10.48550/arXiv.1711.05101.

[30] A. Yadav and S. Yadav , “Enhancing Oral Squamous Cell Carcinoma Detection: A Transfer Learning Perspective on Histopathological Analysis Using ResNet‐18, AlexNet, DenseNet‐169, and DenseNet‐201 With Cyclic Learning Rate,” International Journal of Intelligent Systems and Applications in Engineering 12, no. 17s (2024): 17s.

